# Hispanic Cancer Survivors Exposed to Multiple Natural Disasters: Pre–Post-Disaster Changes in Anxiety, Depression, PTSD, Perceived Stress, and Physical Symptom Burden

**DOI:** 10.3390/ijerph21091237

**Published:** 2024-09-19

**Authors:** Eida M. Castro-Figueroa, Cristina Peña-Vargas, Mónica Rodríguez-Santiago, Juan I. Figueroa, Ruthmarie Hernández, Zindie Rodríguez, Heather Jim, Cristina Pereira, Normarie Torres-Blasco, Idhaliz Flores, Rosario Costas-Muñiz, Guillermo N. Armaiz-Pena

**Affiliations:** 1 Clinical Psychology Department, School of Behavioral and Brain Sciences, Ponce Health Sciences University, Ponce 00716, Puerto Rico; normarietorres@psm.edu; 2 School of Medicine, Ponce Health Sciences University, Ponce 00716, Puerto Rico; 3 School of Medicine, University of Puerto Rico-Medical Sciences Campus, San Juan 00935, Puerto Rico; monica.rodriguez17@upr.edu; 4 Biomedical Sciences Department, School of Medicine, Ponce Health Sciences University, Ponce 00716, Puerto Rico; jifigueroa17@stu.psm.edu (J.I.F.); iflores@psm.edu (I.F.); garmaiz@psm.edu (G.N.A.-P.); 5 Wilmot Cancer Institute, University of Rochester Medical Center, Rochester, NY 14627, USA; ruthmarie_hernandez@urmc.rochester.edu; 6 Moffitt Cancer Center, Tampa, FL 33612, USA; heather.jim@moffitt.org; 7 Battle Creek VA Medical Center, Battle Creek, MI 49037, USA; cpereira14@stu.psm.edu; 8 Memorial Sloan Kettering Cancer Center, New York, NY 10065, USA; costasmr@mskcc.org

**Keywords:** earthquakes, natural disasters, COVID-19 pandemic, cancer symptom burden, psychological distress, PTSD, perceived stress, resilience, social support

## Abstract

Background: Studies evaluating the effects of natural disasters on cancer outcomes are scarce, especially among USA ethnic minority groups, and none have focused on the effects of concurrent natural disasters and the COVID-19 pandemic. The goal of this secondary data analysis is to explore the impact of concurrent exposure to COVID-19 and earthquakes on psychological distress and symptom burden among Puerto Rican cancer survivors. Methods: This secondary data analysis (*n* = 101) was part of a longitudinal case–control cohort study (*n* = 402) aimed at describing unmet psychological needs among Puerto Rican cancer patients and non-cancer subjects previously exposed to Hurricane María in 2017. The research team pooled data from participants (cancer survivors and non-cancer group) from their baseline assessments and from follow-up assessments conducted during January–July 2020 (earthquake and the lockdown period). A descriptive, paired *t*-test, non-parametric mean rank test, and two-sided Pearson correlation analyses were performed. Results: Psychological distress and cancer symptom burden diminished over time. Resilience was significantly correlated with all the psychological and symptom burden variables during both pre- and post-earthquake and COVID-19 assessment periods. Conclusions: The results support the role of resilience, social support, and post-traumatic growth as potential protective factors preventing psychological distress and diminishing cancer symptom burden among cancer survivors exposed to natural disasters and the COVID-19 pandemic.

## 1. Introduction

The Caribbean region, where Puerto Rico is located, is vulnerable to natural disasters due to its location, climate, tropical storms, and seismic activity [1]. On 20 September 2017, Hurricane María (referred to throughout as María), a category 4 hurricane, killed an estimated 2975 people and caused approximately USD 90 billion in property damage as well as widespread devastation (long-term blackouts, extensive structural damages, and food shortages). Approximately two years later, in January 2020, a 6.4 ML earthquake and aftershocks rattled the southwestern region of PR, rendering 8000 people homeless and 40,000 citizens living outside their unsafe, damaged homes with an estimated USD 3.1 billion in damages. Thousands of people in PR were still living outside of their damaged homes when the first COVID-19 case in March 2020 was reported in PR. These tragic circumstances provide a unique context to understand the impact of multiple natural disasters on cancer health outcomes.

Studies evaluating the effects of natural disasters on cancer outcomes are scarce [2,3], including the impact of exposure to multiple disasters in ethnic minority groups with cancer [4], and none have focused on the effects of concurrent natural disasters and the COVID-19 pandemic. A cancer diagnosis and treatment already can be perceived by several cancer patients as a traumatic experience [5,6], considering that it is a life-threatening disease, thus provoking negative emotional reactions such as fear, helplessness, and horror [7]. This pre-existing traumatic experience, which could evolve into post-traumatic stress disorder (PTSD) [8], can be exacerbated after exposure to a natural disaster [9]. For instance, Fernández et al. (2017) [9] explored the effects of pre-disaster PTSD in the development of post-disaster PTSD after an 8.8 earthquake, on the Richter Scale, impacted Chile. They found that 11.5% of participants had pre-disaster PTSD, and 2% had pre- and post-disaster PTSD. Moreover, they found that those developing post-disaster PTSD (10.2%) met the criteria for pre-disaster mood and anxiety mental health disorders.

Natural disasters impact the mental health of those exposed [10], who are more likely to experience high levels of stress, anxiety and depression symptoms, sadness, suicide attempts, sleep disturbance, and changes in appetite [11]. Among cancer patients, natural disasters exert a similar impact on their mental health and negatively affect patients’ quality of life [12]. In summary, there is scientific evidence addressing the effects of single natural disasters on patients’ mental health, but little is known about the potential synergistic effects of combined exposure to multiple natural disasters (e.g., hurricanes, earthquakes, and COVID-19 pandemic) impact on mental health and cancer health outcomes [13,14,15,16,17,18]. The goal of this secondary data analysis is to explore the impact of concurrent exposure to COVID-19 and earthquakes on symptom burden among Puerto Rican cancer survivors exposed to Hurricane María. The team compared pre–post-COVID-19 pandemic and earthquake changes in PTSD, depression, and anxiety symptoms, as well as perceived stress and cancer symptom burden. Also, the team assessed the differences among cancer patients and non-cancer controls regarding the symptoms of anxiety, depression, PTSD, and perceived stress, as well as the role of potentially protective factors such as resilience, post-traumatic growth, social support, and stress management skills. 

## 2. Materials and Methods

This secondary data analysis (*n* = 101) was part of a longitudinal case–control cohort study (cancer-case group and non-cancer control group, *n* = 402, NIH grant #R21MD013674) aimed at describing unmet psychological needs among Puerto Rican cancer patients exposed to Hurricane María, exploring post-María multilevel barriers and facilitators of access to cancer care, and assessing physiological (e.g., circulating markers of stress, hair cortisol, and catecholamines) and psychological markers of stress (e.g., anxiety, depression, and PTSD) linked to exposure to Hurricane María. Participants’ recruitment took place from January 2018 (four months after Hurricane María) to September 2020. Participants were recruited in collaboration with community partners, including local community oncology clinics, cancer support groups, the Puerto Rico American Cancer Society, and community-based cancer support service organizations.

### 2.1. Ethical Statement

This project was revised and approved by the investigators’ academic institution’s Institute Review Board (IRB). It complies with the United States 45 Code of Federal Regulations part 46 (45 CFR 46) related to the Common Rule and human subject regulation.

### 2.2. Participants’ Eligibility and Data Collection Process

For the parent grant, cancer patients were eligible if they were (1) adults [21 years; legal adult age in Puerto Rico]; (2) capable of reading and speaking Spanish; (3) able to provide informed consent; and (4) under the care of their physician for their cancer at the time of post-María critical period (within the first six months after Hurricane María). Participants allocated in the non-cancer control group were eligible if they met eligibility criteria 1, 2, and 3. Also, participants in the control group were matched to the cancer survivor by age, sex, and zip code. Upon signing informed consent, the research assistants conducted baseline assessments. Follow-up assessments were conducted every three months up to a year. For this secondary data analysis, the research team pooled data from cancer patients completing follow-up assessments between September and December 2019. Extracted data included sociodemographic and clinical characteristics, as well as health behaviors, symptoms of depression, anxiety, and PTSD. Then, we compared post-COVID-19 pandemic and earthquake changes with a follow-up assessment conducted between January and July 2020. Data were extracted from the SPSS statistical package data extraction operation function.

### 2.3. Measures

*Post-Traumatic Checklist for DSM-5 (PCL-5)*: The Post-Traumatic Checklist for DSM-5 (PCL-5) is a 20-item questionnaire that corresponds to the 20 criteria for PTSD stated in DSM-5. It contains four subscales corresponding to the four symptom clusters: (1) re-experiencing, (2) avoidance, (3) negative alterations in cognition and mood, and (4) increased arousal and reactivity [19].

*Patient Health Questionnaire-8 (PHQ-8)*: The Patient Health Questionnaire-8 (PHQ-8) is an 8-item questionnaire that measures depressive symptoms as described in the DSM-IV. It uses a four-point scale, with the total score ranging from 0 to 24; a score of 10+ is interpreted as major depression, and a score of 20+ indicates severe major depression [20,21].

*General Anxiety Disorder-7 (GAD-7)*: The General Anxiety Disorder-7 (GAD-7) is a 7-item questionnaire that allows for the rapid detection of generalized anxiety disorder (GAD). It uses a four-point scale with a total score of 0–21; scores of 10+ should be further evaluated, and a score of 15+ represents severe anxiety [20].

*Perceived Stress Scale (PSS)*: The PSS is a 10-item self-report measure of global perceived stress, and its total score ranges from 0 to 40. Higher scores indicate greater levels of perceived stress. The scale has two factors; Factor 1 is considered a “negative” factor and includes six negatively worded items (Items 1, 2, 3, 6, 9, and 10). Factor 2 is considered “positive” and includes four positively worded items (Items 4, 5, 7, and 8) [22,23].

*MD Anderson Symptom Inventory (MDASI)*: The MDASI is a 21-item scale assessing symptom burden in cancer patients. The scale was based on the Brief Pain Inventory and the Brief Fatigue Inventory. Each symptom is rated on an 11-point scale (0–10) to assess the presence and severity of the symptoms in the past 24 h. The MDASI also includes ratings of symptoms’ interference with patients’ daily life activities in the past 24 h [24].

*Brief Resilience Scale (BRS):* This is a self-report measure designed to assess an individual’s ability to bounce back or recover from stress. The BRS specifically focuses on the core aspect of resilience, which is the capacity to recover from adversity. The scale consists of six items, with responses typically rated on a Likert scale ranging from 1 (strongly disagree) to 5 (strongly agree). Higher scores indicate greater resilience [25].

*Interpersonal Support Evaluation List Short Form-12 (ISEL-12)*: The ISEL-12 is a 12-item self-report questionnaire designed to assess perceived social support. The ISEL-12 measures various dimensions of social support, including appraisal support, belonging, support, and tangible support. Each item on the ISEL-12 is rated on a four-point scale (definitely false to definitely true), with higher scores indicating greater perceived social support [26].

*Post-Traumatic Growth Inventory (PTGI)*: This self-report scale is designed to measure positive outcomes that people may experience following a traumatic event. The scale assesses various dimensions of post-traumatic growth, which include personal strength, new possibilities, improved relationships, spiritual growth, and appreciation of life. Each item on the PTGI is rated on a Likert scale, ranging from 0 to 5. Higher scores indicate greater post-traumatic growth [27].

### 2.4. Statistical Analysis

Descriptive statistical analyses were performed to evaluate sociodemographic characteristics from the sample, and chi-square analyses were performed to assess group differences. For the patients’ group, a paired *t*-test was performed to assess the differences in means before and after the earthquake and the pandemic in depression, anxiety, perceived stress, and post-traumatic stress symptoms. To compare mental health outcomes (depression, anxiety, perceived stress, and post-traumatic stress) between patients and controls after the earthquakes and the pandemic, we performed a non-parametric Mann–Whitney test to compare mean ranks, as the sample size between groups did not follow normal distribution. Lastly, to explore the relationship between depression, anxiety, perceived stress, and post-traumatic stress symptoms, with known protective factors (resilience, interpersonal support, post-traumatic stress growth, and the number of stress management skills), two-sided Pearson correlation analyses were conducted. The alpha level used for all the analyses was 0.05, using IBM SPSS software version 29.

## 3. Results

### 3.1. Sociodemographic and Clinical Characteristics and Health Behaviors

Participants’ sociodemographic and clinical characteristics, as well as health behaviors, are summarized in Table 1. Overall, between patients and non-cancer controls, there was a significant difference regarding the household income received not being enough (*p* ≤ 0.05). Most cancer patients reported breast cancer as their primary tumor (36.3%), surgery (75.1%) and chemotherapy (53.1%) as oncology treatment received, and the time since a cancer diagnosis was $\bar{x}$ = 10.44 years. When compared to non-cancer controls ($\bar{x}$ = 3), cancer patients ($\bar{x}$ = 4.37) reported a slightly higher number of multi-morbid chronic conditions. As the abovementioned differences were not statistically significant, we can deduce that cancer patients reported being diagnosed with a mental health illness more frequently than non-cancer control patients. Regarding health behaviors, more cancer patients reported being smokers than non-cancer controls. Lastly, a greater proportion of cancer patients reported engaging in stress management strategies compared to non-cancer controls. Specifically, among cancer patients, the reported practices included meditation (2, 3.6%), yoga (1, 1.8%), listening to relaxing audio (6, 10.9%), watching videos about stress management (1, 1.8%), practicing relaxation techniques independently (6, 10.9%), walking (6, 10.9%), praying (4, 7.3%), practicing hobbies (13, 23.6%), and other stress-reducing activities not specified (3, 6.5%). In contrast, the control group reported the following stress management strategies: meditation (7, 15.2%), listening to relaxing audio (3, 6.5%), watching videos about stress management (1, 2.2%), practicing relaxation techniques independently (3, 6.5%), walking (4, 8.7%), praying (2, 4.3%), engaging in hobbies (4, 8.7%), and other activities (3, 6.5%). This difference in the use of stress management strategies may possibly indicate the unique stressors that cancer patients face and the important effects that these strategies have on their overall well-being.

### 3.2. Pre–Post-Disaster Changes among Cancer Patients

Table 2 summarizes pre–post-earthquake and COVID-19 exposure changes in depression (PHQ-8), anxiety (GAD-7), perceived stress (PSS), PTSD symptoms (PCL-5), and cancer symptom burden (MDASI-total, intensity, and interference). The results reveal that depression symptoms significantly diminished over time. Based on these findings, the team conducted a correlation analysis to explore the relationships among the outcome variables and variables known to serve as protective factors. Table 3 presents the correlation between the outcome variables and known protective factors assessed during the pre-earthquake and COVID-19 exposure (2019). Table 4 summarizes the same correlations for the post-earthquakes and COVID-19 exposure (2020). Resilience and social support were significantly correlated with all the psychological and symptom burden variables during both pre- and post-earthquake and COVID-19 pandemic lockdown assessment periods. Meanwhile, post-traumatic growth was significantly associated with most of the outcome variables during pre-earthquake and COVID-19 pandemic lockdown assessment (see Table 3 and Table 4).

### 3.3. Comparison of Cancer Patients and Non-Cancer Controls 

The team explored the differences among cancer patients and non-cancer controls regarding the symptoms of anxiety, depression, PTSD, and perceived stress (Table 5). The analyses showed no significant differences between the groups.

## 4. Discussion

This study is unique as it provides pre- and post-pilot data on the potential impact of concurrent exposure to earthquakes and the COVID-19 pandemic in a sample of underserved Hispanic cancer patients living in Puerto Rico. The findings reveal that depression symptoms among cancer patients diminished during the post-disaster assessment period. The observed decrease in psychological symptoms during post-disaster assessment may partly be due to the use of antidepressants and/or psychological adaptation [28,29]. Accordingly, psychological symptoms may diminish over time as patients adjust or adapt to their condition and treatment regimen. However, future studies should explore whether the presence or absence of certain mitigating factors before a disaster can influence mental health outcomes after the disaster. The results may also suggest that resilience and social support might have played a role in these positive findings, including cancer symptom burden. One fact potentially explaining these findings is that our sample is unique as cancer survivors were recruited from support-service programs and thus already were part of a social support network, which has been linked to post-disaster exposure resilience outcomes [30]. Also, the majority reported using stress management skills. 

Resilience is a multidimensional construct in which personal (e.g., coping mechanisms), biological (e.g., oxytocin hormone), and environmental factors (e.g., social support and religion) interact to overcome stress and adversity [31,32,33], especially after exposure to natural disasters. There are considerable scientific data evidencing the protective role of resilience and social support in adversity and psychological distress (anxiety and depression) [31,32,33] and PTSD [34], as well as among cancer patients [35,36]. For instance, resilience among cancer patients is associated with a higher quality of life [29] and lower symptoms of depression and anxiety [37]. Another study found that resilience moderated the relationship between stress and psychological distress (anxiety and depression) among prostate cancer patients, and it also mediated the relationship between masculine identity threat, psychological distress, and negative affect [38]. Also, a systematic review conducted by Lau et al. [39] found that higher resilience among cancer patients was associated with improved mental health and quality of life. 

An unexpected finding worth highlighting is the potential role of resilience in cancer symptom burden. There are a handful of studies reporting similar results in cancer patients but none in the context of exposure to natural disasters. For example, one research study concluded that resilience mediated the relationship between cancer symptom burden and quality of life [40]. Another found that resilience moderates the effects of depressive symptoms on fatigue [41]. Also, among breast cancer patients, another study found that higher psychological resilience was associated with lower pain symptom interference; however, the association was weaker among patients presenting high anxiety symptoms. 

One limitation of our study is the high prevalence of lifetime mental health diagnoses in both the cancer patient group (38%) and the control group (33%). This may have obscured differences in mental health outcomes between the groups. Future studies should explore the types of psychopathology diagnoses among participants and consider using a sample without prior mental health diagnoses to provide clearer insights into the specific impact of cancer on mental health outcomes following natural disasters. This study focused on comparing individuals with or without a cancer diagnosis rather than matching control groups based on the absence of mental health issues, thus potentially introducing bias. Another limitation of this secondary data analysis is that the small sample size may limit the ability to detect statistically significant differences. As mentioned in the Methods section, this is a secondary data analysis study derived from a larger observational longitudinal study documenting the multilevel barriers and facilitators of access to cancer care post-Hurricane María that also assessed physiological (e.g., circulating markers of stress, hair cortisol, and catecholamines) and psychological markers of stress (e.g., anxiety, depression, and PTSD) linked to exposure to Hurricane María. When the 2020 earthquakes and the COVID-19 pandemic occurred, our team was conducting follow-up data from the parent grant, and the sample obtained for this secondary analysis was selected from the completed follow-up assessment obtained during that period. Yet, considering the unique circumstances of having a natural disaster co-occurring with the COVID-19 pandemic, and the availability of pre-disaster and COVID-19 pandemic data for exploring their impact on psychological distress and cancer symptom burden, the data obtained from this study are very valuable and promising. As there are very few published studies suggesting a link between resilience and symptom burden in the context of exposure to natural disasters and the potential positive implications of this link, we recommend expanding this line of research further.

## 5. Conclusions

In summary, the data from this pilot study support the potential role of resilience and social support as protective factors preventing psychological distress and diminishing cancer symptoms among cancer survivors. These findings provide a new and interesting perspective on cancer symptom burden research that can be translated into clinical practice. We recommend conducting multidimensional studies elucidating the role of resilience on cancer symptom burden and specific cancer symptoms. Studies with a larger sample size are needed to corroborate these findings, especially the role of resilience in potentially mediating or moderating cancer symptom burden. 

## Figures and Tables

**Table 1:** Participants’ sociodemographic/clinical characteristics and health behaviors.

Variables	Patients, *n* = 55 Mean (SD)/N (%)	Controls, *n* = 46 Mean (SD)/N (%)
***Socio-Demographic Characteristics***		
Age	58.83 (11.44)	58.55 (10.62)
Sex (female)	45 (81.8%)	38 (82.6%)
Civil Status (married)	19 (34.5%)	25 (54.3%)
Employment status (employed)	9 (16.3%)	17 (36.9%)
Income (≤12,001–19,000)	42 (76.3%)	30 (65.2%)
Income enough (yes)	11 (20%)	20 (43.4%) *
Education level (high school or higher)	49 (89%)	41 (89.1%)
***Clinical Characteristics***		
Type of cancer		
Breast	20 (36.3%)	
Uterus	8 (14.5%)	
Thyroid	5 (9%)	
Prostate	3 (5%)	
Others	17 (30%)	
Metastasis (yes)	10 (18.1%)	
Cancer treatment		
Radiation therapy	19 (34.4%)	
Chemotherapy	33 (53.1%)	
Surgery	44 (78.1%)	
Hormone therapy	6 (15.6%)	
Time since cancer diagnosis (years)	10.44 (10.60)	
Self-reported body max index (BMI)	31.72 (7.81)	
Chronic conditions comorbidity	4.37 (2.48)	3 (2.41)
Lifetime mental health diagnosis	21 (38.1%)	15 (32.6%)
***Health Behaviors***		
Use of antidepressants	14 (70%)	6 (30%)
Sleep hours per day (>8 h per day)	38 (69%)	33 (71.7%)
Smoking behavior (yes)	6 (10.9%)	4 (8.6%)
Alcohol consumption (yes)	17 (30.9%)	16 (34.7%)
Physical activity (yes)	31 (56.3%)	28 (60.8%)
Stress management strategies (yes)	32 (58.1%)	22 (47.8%)

* *p* ≤ 0.05.

**Table 2:** The mean changes and effect size during pre–post-earthquake and COVID-19 exposure considering outcome variables’ data (*n* = 55).

Variables	Pre (Mean/SD)	Post (Mean/SD)	t	Cohen’s d	Sig.
MDASI—Intensity	39.09 (35.72)	36.65 (31.19)	0.633	0.085	0.529
MDASI—Interference	20.07 (18.47)	16.91 (18.06)	1.555	0.212	0.126
MDASI—Total	59.17 (51.99)	52.76 (46.34)	1.222	0.166	0.227
PHQ-8	8.76 (6.50)	6.87 (6.44)	2.492	0.336	0.016
GAD-7	7.95 (6.49)	6.89 (6.19)	1.329	0.179	0.190
PSS	16.58 (9.09)	15.87 (8.37)	0.605	0.082	0.548
PCL-5	24.44 (18.71)	20.15 (17.99)	1.801	0.245	0.077

**Table 3:** Pre-earthquake and COVID-19 exposure correlations among the outcome variables and known protective factors (*n* = 55).

	Depression (PHQ-8)	Anxiety GAD-7	Perceived Stress (PSS)	PTSD (PCL-5)	SymptomBurden MDASI	Symptom Intensity MDASI	Symptom Interference MDASI
Resilience (BRB)	−0.637 **	−0.671 **	−0.755 **	−0.658 **	−0.425 **	−0.486 **	−0.470 **
Social support (ISEL)	−0.315 *	−0.310 *	−0.425 **	−0.355 **	−0.336 *	−0.380 **	−0.368 **
Post-traumatic growth (PTGI)	0.205	0.273 *	0.325 *	0.325 *	0.410 **	0.377 **	0.423 **
Stress management skills	−0.084	0.220	0.342	0.376 *	0.106	0.104	0.062

** *p* ≤ 0.01; * *p* ≤ 0.05.

**Table 4:** Post-earthquake and COVID-19 exposure correlations among the outcome variables and known protective factors (*n* = 55).

	PHQ-8	Gad-7	PSS	PCL-5	MDA-SymptomBurden	MDA-Intensity	MDA-Interference
Resilience (BRB)	−0.608 **	−0.705 **	−0.666 **	−0.679 **	−0.633 **	−0.611 **	−0.662 **
Social support (ISEL)	−0.412 **	−0.374 *	−0.325 *	−0.329 *	−0.300 *	−0.400 *	−0.356 *
Post-traumatic growth (PTGI)	0.152	0.091	0.022	0.199	0.288*	0.134	0.246
Number of stress management skills	0.324	0.234	0.103	0.232	0.093	0.082	0.101

** *p* ≤ 0.01; * *p* ≤ 0.05.

**Table 5:** Comparison of patients (*n* = 55) and controls (*n* = 46) during the earthquake and COVID-19 exposure period.

Variables	Code	Mean Rank	Sum of Ranks	Z	Sig.
PHQ-8	Control	48.74	2242.00	−0.858	0.391
	Patient	53.77	3011.00		
GAD-7	Control	48.26	2220.00	−1.008	0.314
	Patient	54.16	3033.00		
PSS	Control	46.89	2157.00	−1.428	0.153
	Patient	55.29	3096.00		
PCL-5	Control	49.12	2259.50	−0.738	0.461
	Patient	53.46	2993.50		

## Data Availability

The data presented in this study will be available upon request due to time and technical limitations.
